# Automated home-cage for the evaluation of innate non-reflexive pain behaviors in a mouse model of inflammatory pain

**DOI:** 10.1038/s41598-021-91444-4

**Published:** 2021-06-10

**Authors:** Hasriadi, Peththa Wadu Dasuni Wasana, Opa Vajragupta, Pornchai Rojsitthisak, Pasarapa Towiwat

**Affiliations:** 1 Pharmaceutical Sciences and Technology Program, Faculty of Pharmaceutical Sciences, Chulalongkorn University, Bangkok, 10330 Thailand; 2 Research Affairs, Faculty of Pharmaceutical Sciences, Chulalongkorn University, Bangkok, 10330 Thailand; 3 Department of Food and Pharmaceutical Chemistry, Faculty of Pharmaceutical Sciences, Chulalongkorn University, Bangkok, 10330 Thailand; 4 Natural Products for Ageing and Chronic Diseases Research Unit, Chulalongkorn University, Bangkok, 10330 Thailand; 5 Department of Pharmacology and Physiology, Faculty of Pharmaceutical Sciences, Chulalongkorn University, Bangkok, 10330 Thailand

**Keywords:** Experimental models of disease, Preclinical research

## Abstract

The failure to develop analgesic drugs is attributed not only to the complex and diverse pathophysiology of pain in humans but also to the poor experimental design and poor preclinical assessment of pain. Although considerable efforts have been devoted to overcoming the relevant problems, many features of the behavioral pain assessment remain to be characterized. For example, a decreased locomotor activity as a common presentation of pain-like behavior has yet to be described. Studies on mice experimentally induced with carrageenan have provided opportunities to explore pain-related behaviors in automated home-cage monitoring. Through this approach, the locomotor activities of mice with carrageenan-induced inflammatory pain can be precisely and objectively captured. Here, we found that the mobile behaviors of mice reduced, and their immobility increased, indicating that carrageenan induction in mice caused a significant decrease in locomotor activity. These non-reflexive pain behaviors were strongly correlated with the reflexive pain behaviors measured via von Frey and plantar tests. Furthermore, the pharmacological intervention using indomethacin improved the locomotor activity of mice with carrageenan-induced pain. Thus, the analysis of the locomotor activity in automated home-cage monitoring is useful for studying the behavioral analgesia and the pharmacological screening of analgesic drugs. The combined evaluation of reflexive and non-reflexive pain behaviors enhances the translational utility of preclinical pain research in rodents.

## Introduction

Inflammatory pain is a complex pathophysiological condition caused by inflammation due to a tissue injury or inflammation-associated diseases^1^. This type of pain is characterized by the excessive release of inflammatory mediators, such as prostaglandin E2 (PGE2), cytokines, chemokines, and growth factors^2^. Although many analgesic drugs have been used in clinical settings to treat inflammatory pain, the adverse effects of these drugs warrant the finding of new analgesic drugs with an enhanced efficacy and fewer adverse effects.

Although many compounds have been screened in terms of their analgesic activity in clinical trials, only 11% of these compounds have been approved as analgesic drugs^3^. One of the reasons why analgesics drug candidates fail in clinical trials is their poor preclinical design, including poor behavioral pain assessment^4^. Most of the current efforts in preclinical studies involving animals are based primarily on reflexive pain assessment, which does not fully mimic the clinical features of pain^5^. Conventional pain assessments, such as evoked pain tests, rely only on the sensory component of pain in rodents. However, this approach has limitations, such as handling-induced stress, inefficiency, time- and labor-consuming steps, inconsistent results, and subjective impression measurement^6–8^. Consequently, it can provide false positive/negative results. Evoked pain tests also have obvious limitations because human pain behaviors are primarily characterized by spontaneous pain (cognitive processes, decision making, attention, motivation)^9,10^. The assessment of evoked pain behaviors only yields a limited understanding of complex behavioral pain. The classical preclinical models of reflexive pain have proven the effectiveness of several FDA-approved drugs in pharmacological screening. However, numerous other entities, including Tachykinin NK1 receptor antagonists, fatty acid amide hydrolase (FAAH) inhibitors, losmapimod (p38 MAP kinase inhibitor), and PF‐05089771 (Nav1.7 inhibitor), which have been proven to be effective in preclinical trials failed in clinical trials^11–15^. Recent progress in pain research suggests important alternatives to pain assessment to overcome these problem. Therefore, the assessment of pain has significantly increased through non-reflexive pain behaviors, including weight-bearing, burrowing, gait analysis, facial grimace scale, conditioned place preference test, open-field test, and automated home-cage monitoring^6,7^.

Locomotor activity, one of the non-evoked pain measures, has been utilized as an indicator of behavioral analgesia in numerous pain models, such as complete Freund's adjuvant (CFA)-induced arthritis^16^, postoperative pain^17^, spinal cord injury^18,19^, pruritus^20^, streptozotocin-induced diabetic neuropathy, and carrageenan-induced acute inflammatory pain^21,22^. In these animal models of pain, locomotor activity is improved by the administration of standard analgesic drugs. Moreover, the evaluation of locomotor activity in rodents has a translational value to pain in humans, where patients with pain also exhibit physical disability^23,24^. In addition, clinical studies have demonstrated the correlation of improved physical disability with the improved well-being and quality of life of patients experiencing pain^25^.

Several studies have applied automated behavioral analysis, including home-cage monitoring, to measure the locomotor activity of rodents^26–30^. For instance, the automated home-cage monitoring, Laboratory Animal Behavior Observation, Registration and Analysis System (LABORAS), has been introduced in preclinical studies to assess non-reflexive pain behaviors^31–34^. The successive generation of non-evoked pain behaviors in the LABORAS system has been observed in several rodent models of pain, including CFA^31^, collagen-induced arthritis (CIA)^32,35,^ and surgical destabilization of the medial meniscus model of osteoarthritis^34^. Moreover, the correlation between evoked and non-evoked pain behaviors has been demonstrated in CFA and CIA models^31,35^. Conversely, not all pain models exhibited non-evoked pain behaviors in the LABORAS system as observed in mouse models of partial meniscectomy (PMX) and spared nerve injury (SNI)^33,32^. The use of automated home-cage monitoring is conceptually appealing because it can provide a home-like and less stressful environment to animals. Stress can be a confounding factor because it induces either hyperalgesia or analgesia in rodents^36,37^. Through this automated behavioral system mouse behaviors with a comprehensive and accurate behavioral phenotype can be automatically analyzed to capture the complexity of animal behaviors, characterize the timeframe in detail, reduce animal stress, minimize experimental bias, and decrease environmental-dependent variabilities^38^. Furthermore, the use of automated home-cage monitoring minimizes human interaction thereby reducing the influence of human-related factors, including the gender of experimenters, experience on handling rodents, and prior contact with rodents, on their behavior^39^. However, in the LABORAS system, mice are assessed individually in a cage wherein their physiological condition may be affected by isolation because they are social creatures.

As stated before, preclinical experiments, including behavioral assessments, should be improved to characterize the human condition of pain in rodents, but this trait has not been sufficiently captured by conventional methods. Hence, in this study, the automated home-cage monitoring LABORAS was used to assess the short- and long-term locomotor activities of carrageenan-induced mice, an acute model of inflammatory pain. However, several non-evoked pain behaviors, including short-voluntary movement in the cage^22^, exploratory behavior in the open-field^21^, weight-bearing and gait characteristics^40^, and grimace score^41^ have been characterized in rodents induced by carrageenan. However, studies have yet to evaluate the innate behaviors of carrageenan-induced mice in the LABORAS system and their correlation with evoked pain behaviors. Hence, this study is the first to demonstrate the impaired locomotive behaviors induced by carrageenan in the LABORAS home-cage system. Overall, this study provides evidence supporting the possibility of using the innate behaviors of mice induced by carrageenan as an alternative behavioral indicator of pain.

## Results

### Induction of thermal and mechanical sensitivity by carrageenan challenge

**Figure 1:**
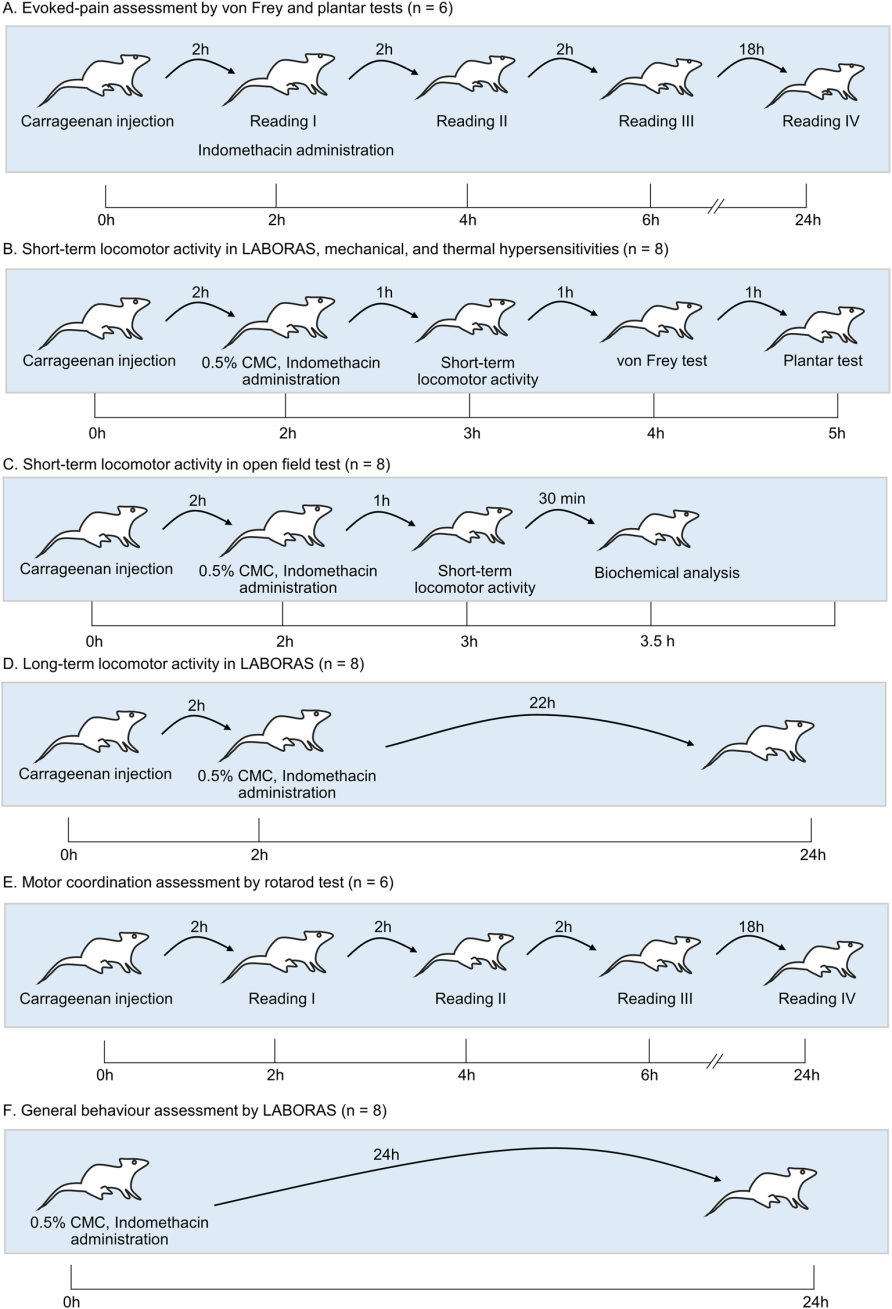
The schematic illustration of the experimental design and groups.

**Figure 2:**
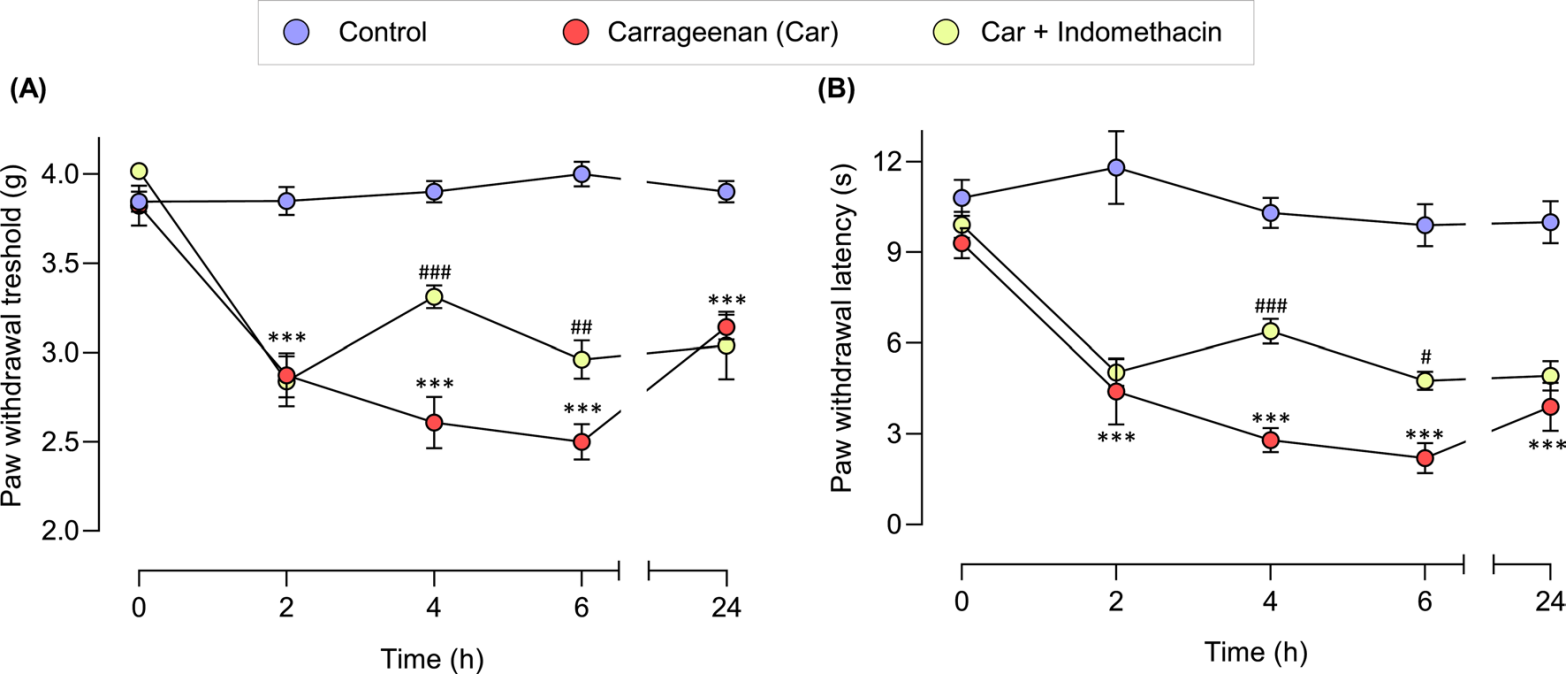
Carrageenan-treated mice exhibited increased mechanical and thermal sensitivities. The paw withdrawal threshold to mechanical stimuli (**A**) and paw withdrawal latency to thermal stimuli (**B**). Data are presented as mean ± SEM for n = 6/group. ***p < 0.001 compared to the vehicle-treated group and ^#^*p* < 0.05, ^##^*p* < 0.01 and ^*###*^*p* < 0.001 compared to the carrageenan-induced group.

Mice were administered with an intraplantar injection of carrageenan and evoked pain behaviors were assessed at 2, 4, 6, and 24 h post-carrageenan administration (Fig. 1A). Carrageenan-induced mice demonstrated thermal and mechanical hypersensitivities in the ipsilateral paw compared with those in the control group. These hypersensitivities initially occurred 2 h post-drug administration and peaked at 6 h post-carrageenan administration. They were maintained up to 24 h post-carrageenan challenge (Fig. 2). Indomethacin, administered 2 h after the carrageenan induction improved the paw withdrawal threshold and latency at 2 and 4 h post-drug administration. The effect of indomethacin peaked at 2 h post-drug administration, declined at 4 h, and no effect was observed at 22 h post-drug administration (Fig. 2).

### Behavioral characterization of LABORAS automated home-cage monitoring: locomotive parameters, composition, and correlations of the related parameters

**Figure 3:**
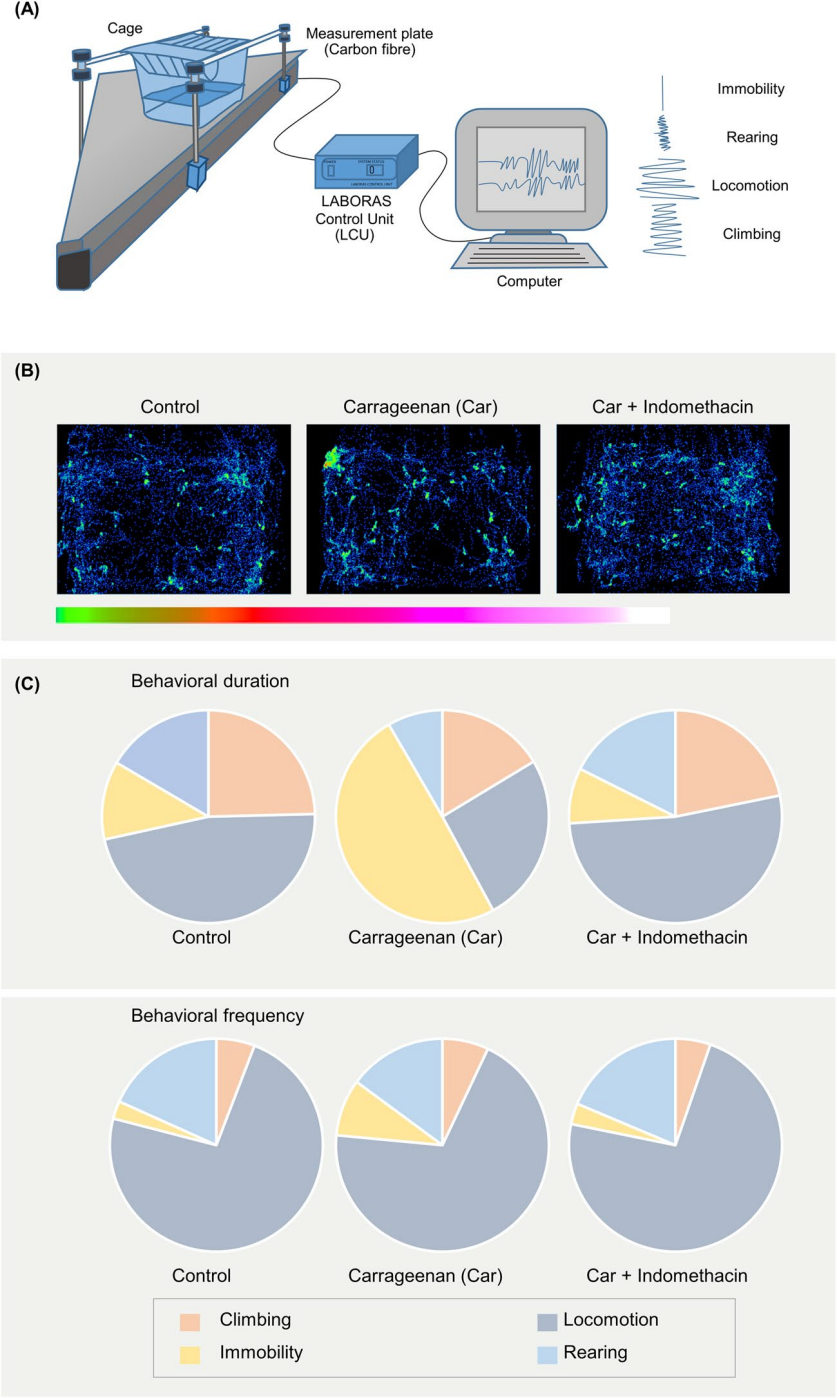
(**A**) Schematic representation of experimental set-up (**B**) position distribution of a single mouse in the cage, (**C**) behavioral composition of locomotor activity (undefined behaviors are excluded from analysis).

A LABORAS automated system was used to test the locomotive behaviors of the mice precisely and comprehensively. This system can generate several locomotive behaviors, such as duration and frequency of climbing, locomotion, rearing, immobility, distance traveled, and average speed (Fig. 3A). On the test day, the mice treated with the control, carrageenan, or carrageenan + indomethacin were allowed to be in the automated home-cage, and their behaviors were automatically recorded using the LABORAS (Fig. 1B). The locomotor activity was evaluated for 30 min, which is a commonly used duration for classical locomotion tests (e.g., open-field test). Through this system, the position distribution of the mice in the cage could be visualized (Fig. 3B). As shown in the position distribution, the density of movement of the carrageenan-induced group was lower than that of the control group. However, after the treatment with indomethacin, the density of the track improved. In terms of the composition of locomotive behaviors in short-term locomotor activity, the control group of the mice spent 24.9 ± 4.6%, 47.1 ± 2.6%, 11.3 ± 4.1% and 16.7 ± 1.5% of the time on climbing, locomotion (walking and running), immobility, and rearing, respectively. By comparison, the frequencies of these behaviors were 5.7 ± 0.9%, 73.3 ± 1.5%, 2.64 ± 0.38% and 18.35 ± 1.63% for climbing, locomotion (walking and running), immobility and rearing, respectively. In the carrageenan-treated group, the mice spent 17.1 ± 4.0%, 28.2 ± 3.4%, 45.9 ± 6.5%, and 8.7 ± 0.8% of the time on climbing, locomotion, immobility, and rearing, respectively, and their frequencies were 6.8 ± 0.9%, 69.8 ± 11.4%, 8.5 ± 0.9%, and 14.8 ± 1.0%, respectively. In the carrageenan + indomethacin-treated group, the mice spent 21.2 ± 3.0%, 52.3 ± 1.7%, 8.3 ± 3.1%, and 18.1 ± 1.8% of the time on climbing, locomotion, immobility, and rearing, respectively, and their frequencies were 5.2 ± 0.9%, 72.8 ± 8.1%, 3.4 ± 1.2%, and 18.6 ± 1.1%, respectively (Fig. 3C). In the present study, undefined behaviors of behavioral duration (66.3 ± 1.6%) and frequency (44.7 ± 0.3%), were excluded from analysis. Hence, the administration of carrageenan reduced the short-term locomotor activity of mice compared with that of the control group and the administration of indomethacin reversed the impaired locomotor activity induced by carrageenan.

### Impairment of exploratory behaviors by carrageenan challenge

**Figure 4:**
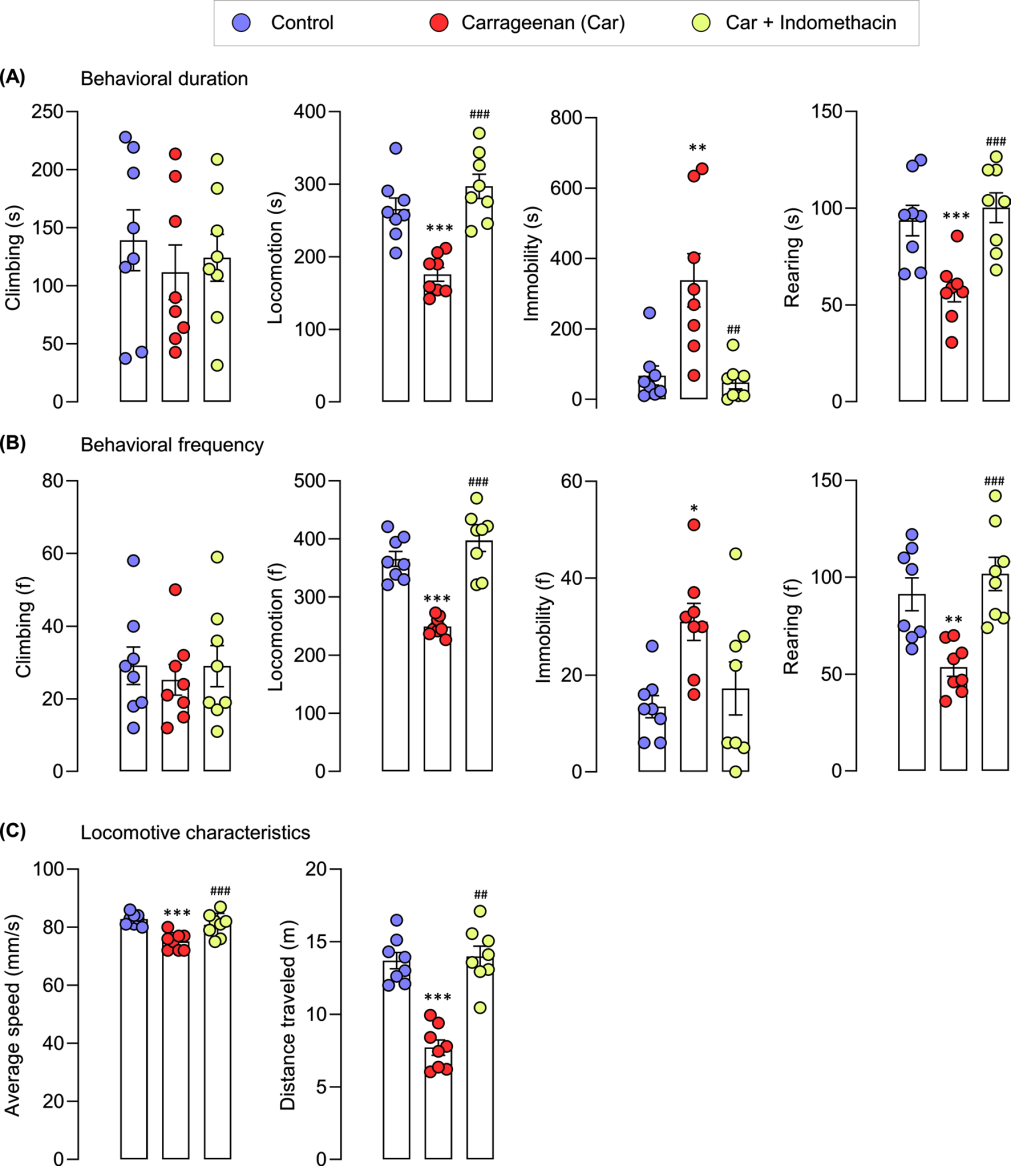
Locomotor activity driven by exploratory behavior of control mice, carrageenan mice, carrageenan + indomethacin mice in automated home-cage LABORAS. Data are expressed as means ± SEM from 8 mice in each group. *, **, *** denote significant difference compared to the control group at p < 0.05, p < 0.01, and p < 0.001, respectively. ^##^ and ^###^ denote significant difference compared to indomethacin-treated group at p < 0.01, p < 0.001, respectively.

Exploratory behaviors (short-term) in automated home-cage monitoring were investigated to test whether the locomotor activity of carrageenan-induced mice was impaired (Fig. 1B). The time spent by carrageenan-induced mice on mobile behaviors (rearing and locomotion) was significantly shorter than that spent by the control mice. Furthermore, the time spent by carrageenan-induced mice in immobility was longer than that of the control mice (Fig. 4A). Moreover, carrageenan-induced mice demonstrated significantly less mobile behaviors, greater immobility, significantly shorter distance traveled and slower average speed than the control mice did (Fig. 4B,C). In addition, during the first 0–5 min, carrageenan slightly affected locomotive behaviors compared with those of the vehicle-treated mice. Slight changes could be observed at this time point, possibly because of the effects masked by their exploratory behaviors in the cage. A substantial difference was observed at 5–10 min of measurement, as indicated by the significant difference in the duration and frequency of locomotion, and distance traveled between the vehicle- and carrageenan-treated groups (Supplementary Fig. S1).

These results suggested that the locomotor activity driven by exploratory behaviors of carrageenan-induced mice decreased. Moreover, the treatment with indomethacin reversed the locomotive impairment induced by carrageenan (rearing, locomotion, average speed, and distance traveled) to the same level as the control group (Fig. 4). These results indicated that indomethacin could effectively enhance the exploratory behaviors impaired by carrageenan administration in LABORAS automated home-cage monitoring.

**Figure 5:**
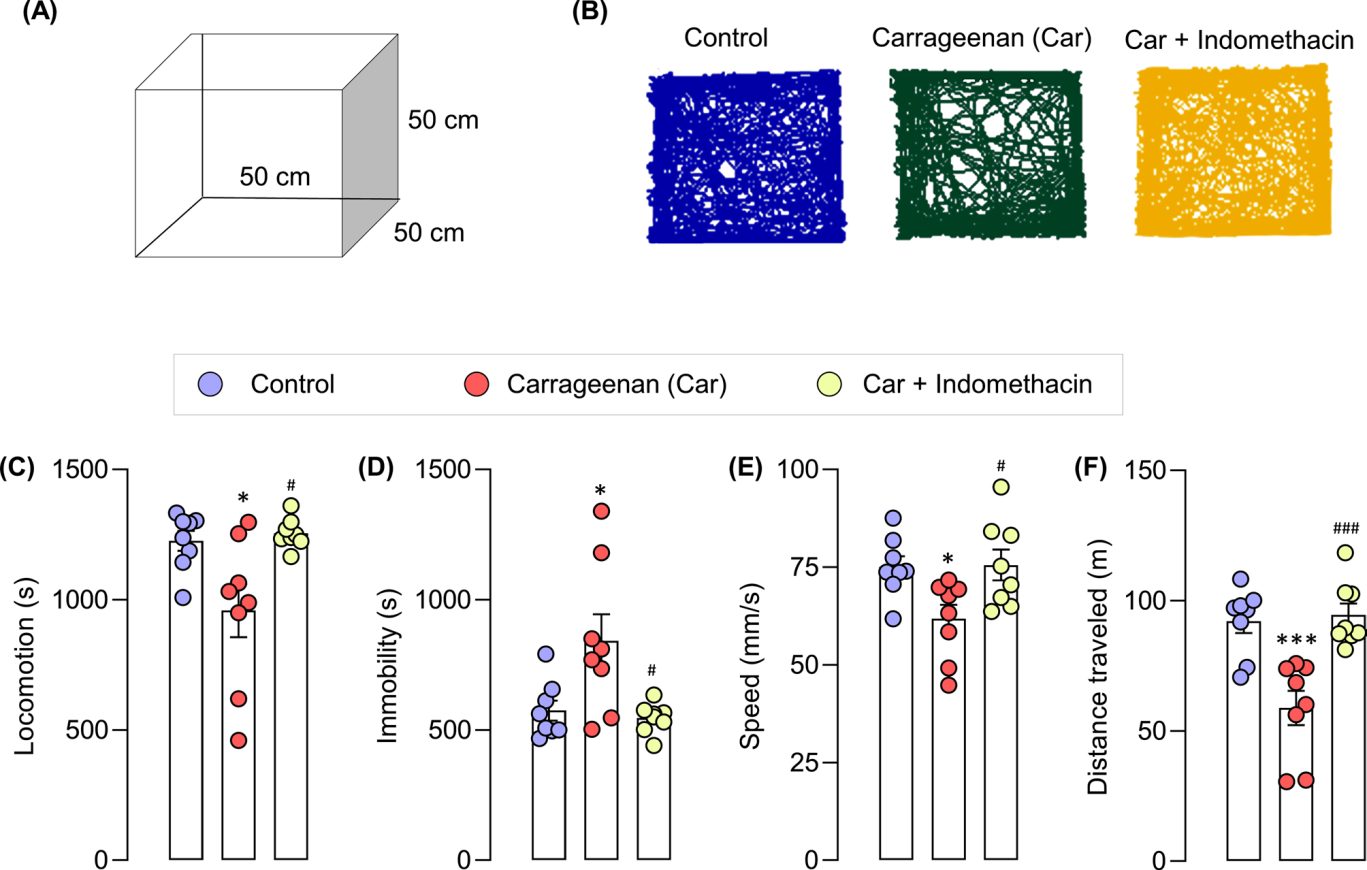
Locomotor activity driven by exploratory behaviors of control mice, carrageenan mice, carrageenan + indomethacin mice in the open-field test. Data are expressed as means ± SEM from 8 mice in each group. *, *** denote significant difference compared to the control group at p < 0.05, and p < 0.001, respectively. ^#^ and ^###^ denote significant difference compared to indomethacin-treated group at p < 0.05, p < 0.001, respectively.

Exploratory behaviors were analyzed (30 min) using the open-field test to compare the LABORAS system and the open-field test (Figs. 1C, 5A). Similar to the results of the LABORAS, the findings of the open-field test showed the increased immobility and reduced locomotion, speed and distance traveled in carrageenan-induced mice (Fig. 5B–F). These results indicated that the intraplantar administration of carrageenan impaired the locomotor activity. Indomethacin treatment improved the carrageenan-induced locomotor impairment (Fig. 5). However, mice spent more time on locomotion and immobility and traveled in the open-field test more than they did the LABORAS monitoring.

### Correlation between evoked pain behaviors and locomotor activity

**Figure 6:**
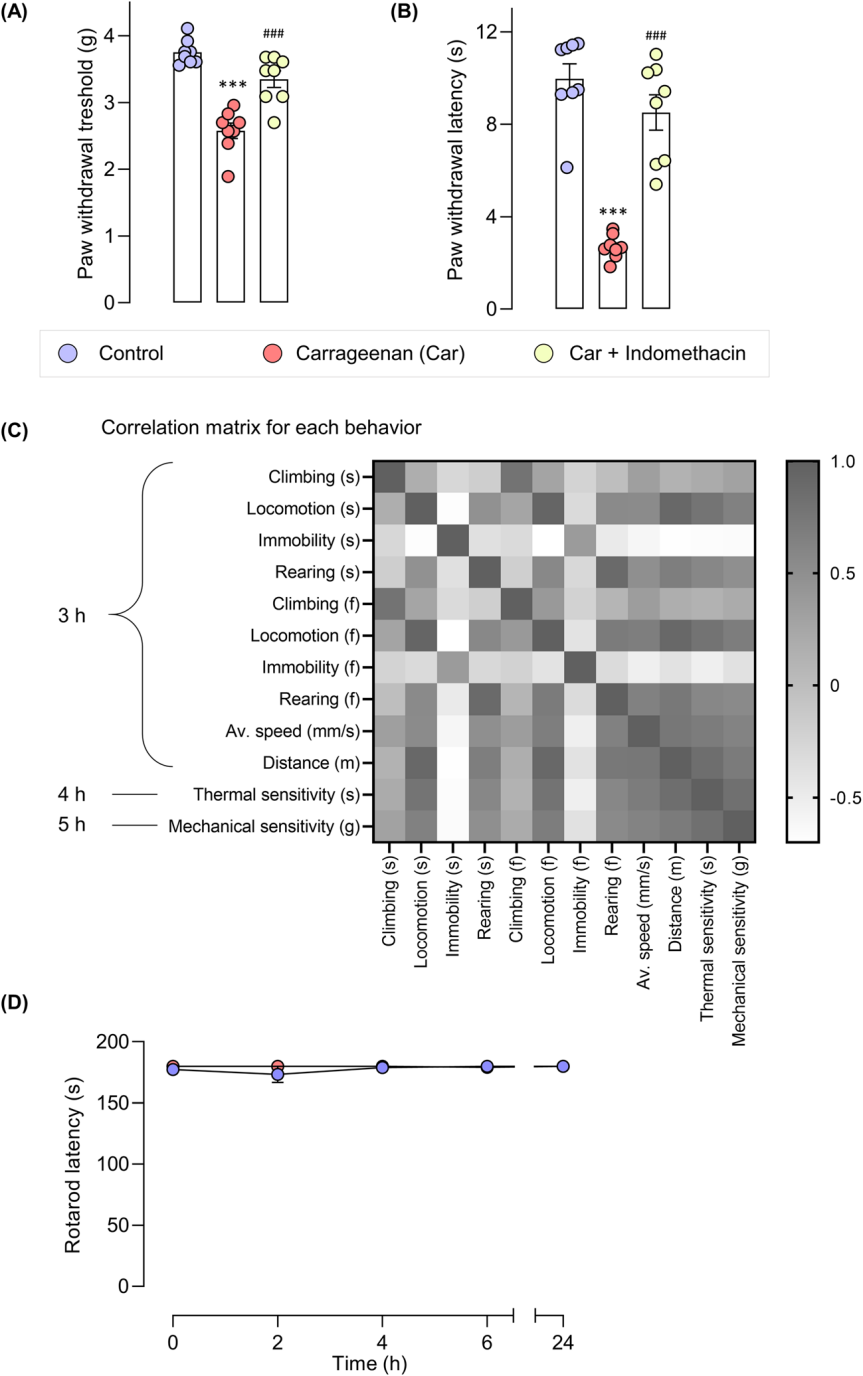
Indomethacin improved the carrageenan-induced mechanical (**A**) and thermal hypersensitivities (**B**). The effect of carrageenan induction on motor coordination in mice (**C**), and the correlation between each locomotive behavior in automated home-cage and thermal and mechanical hypersensitivities (**D**). *** denotes significant difference at p < 0.001 (control vs carrageenan). ^###^ denotes significant difference at p < 0.001 (carrageenan and carrageenan + indomethacin).

In line with the increased thermal and mechanical hypersensitivities, the locomotor activity of the carrageenan-induced mice was impaired in the LABORAS home-cage monitoring. These behaviors were improved by indomethacin administration (Fig. 6A,B). The correlation between evoked pain behaviors and locomotor activity quantified in the LABORAS system was determined via Pearson correlation to predict the potential measures of behavioral pain in the carrageenan-induced mouse model (Fig. 6C). The data for correlation analysis were extracted from the control, carrageenan, and indomethacin + carrageenan groups. The correlation analysis of the behaviors showed that mobile behaviors (climbing, rearing, locomotion) were positively correlated with one another. These mobile behaviors were also positively correlated with the distance traveled and average speed. Conversely, they were negatively correlated with immobility. The correlation analysis between locomotor activity and evoked pain behaviors (thermal and mechanical sensitivity) revealed that the paw withdrawal latency and threshold values were positively correlated with the mobile behaviors (climbing, rearing, and locomotion). By contrast, they were negatively correlated with immobility.

### Effect of carrageenan induction on motor coordination in mice

A rotarod test was performed to examine whether the decreased locomotor activity observed in home-cage monitoring was due to impaired motor coordination (Fig. 1E). The results demonstrated no significant difference between the control and carrageenan-treated groups at 2, 4, 6, and 24 h of measurements (Fig. 6D).

### Impairment of the long-term locomotor activity by carrageenan challenge

**Figure 7:**
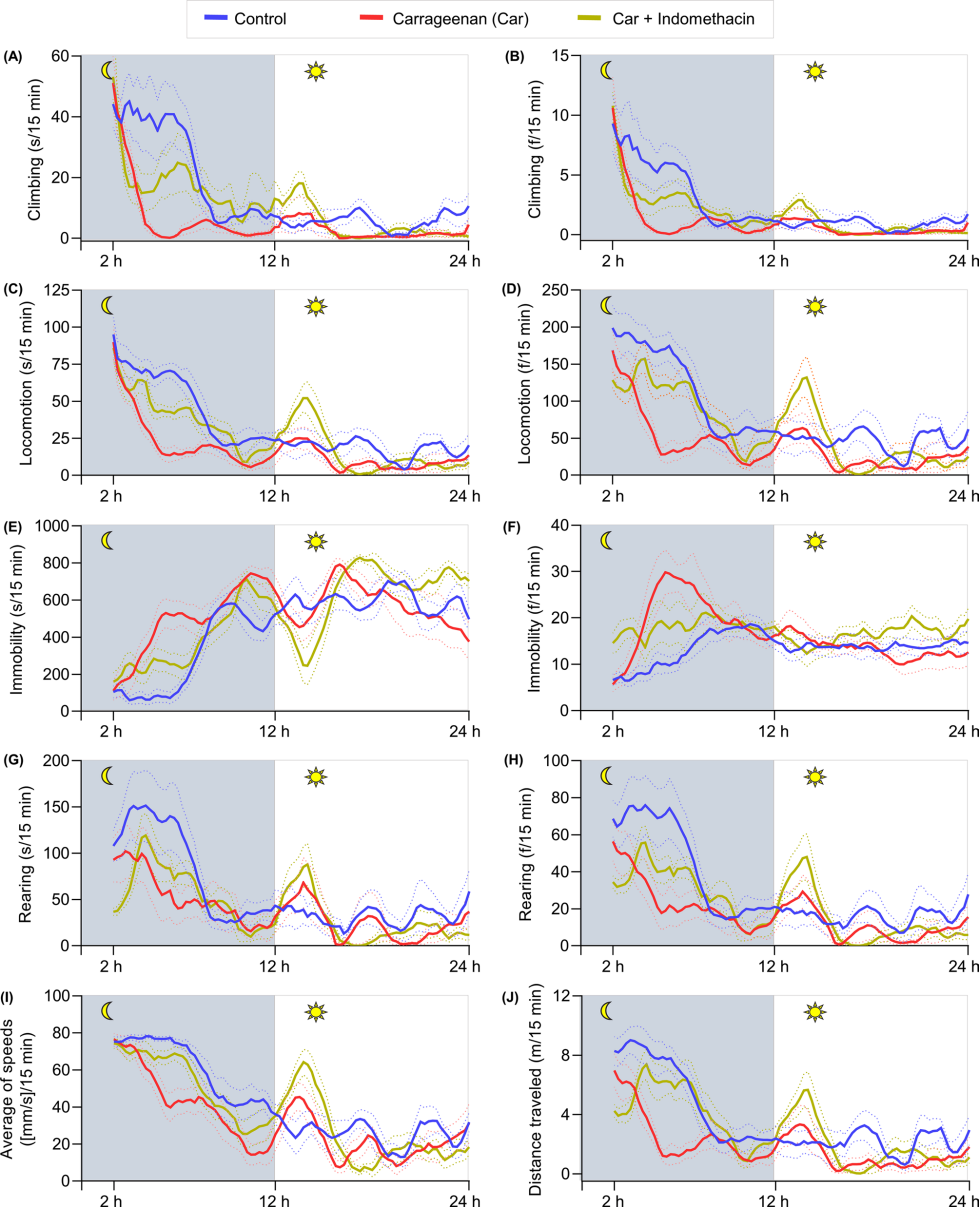
Time-series analysis of the long-term locomotor activity of control-, carrageenan-, carrageenan + indomethacin-treated mice in automated home-cage LABORAS. Data represent a 2 h moving average for the mean locomotion time of mice in each 15 min. Solid and dashed lines represent the mean and SEM, respectively.

The time-series analysis of long-term locomotor activities was demonstrated using line graphs (Fig. 7). Mice are nocturnal rodents, so they are more active at night. This behavior is characterized by the overall increased time and frequency of mobile behaviors and decreased immobility observed at night compared with that during daytime. The locomotive behaviors of the control and carrageenan groups during daytime slightly differed. At nighttime, the duration and frequency of climbing, frequency of locomotion, and distance traveled significantly differed.

**Figure 8:**
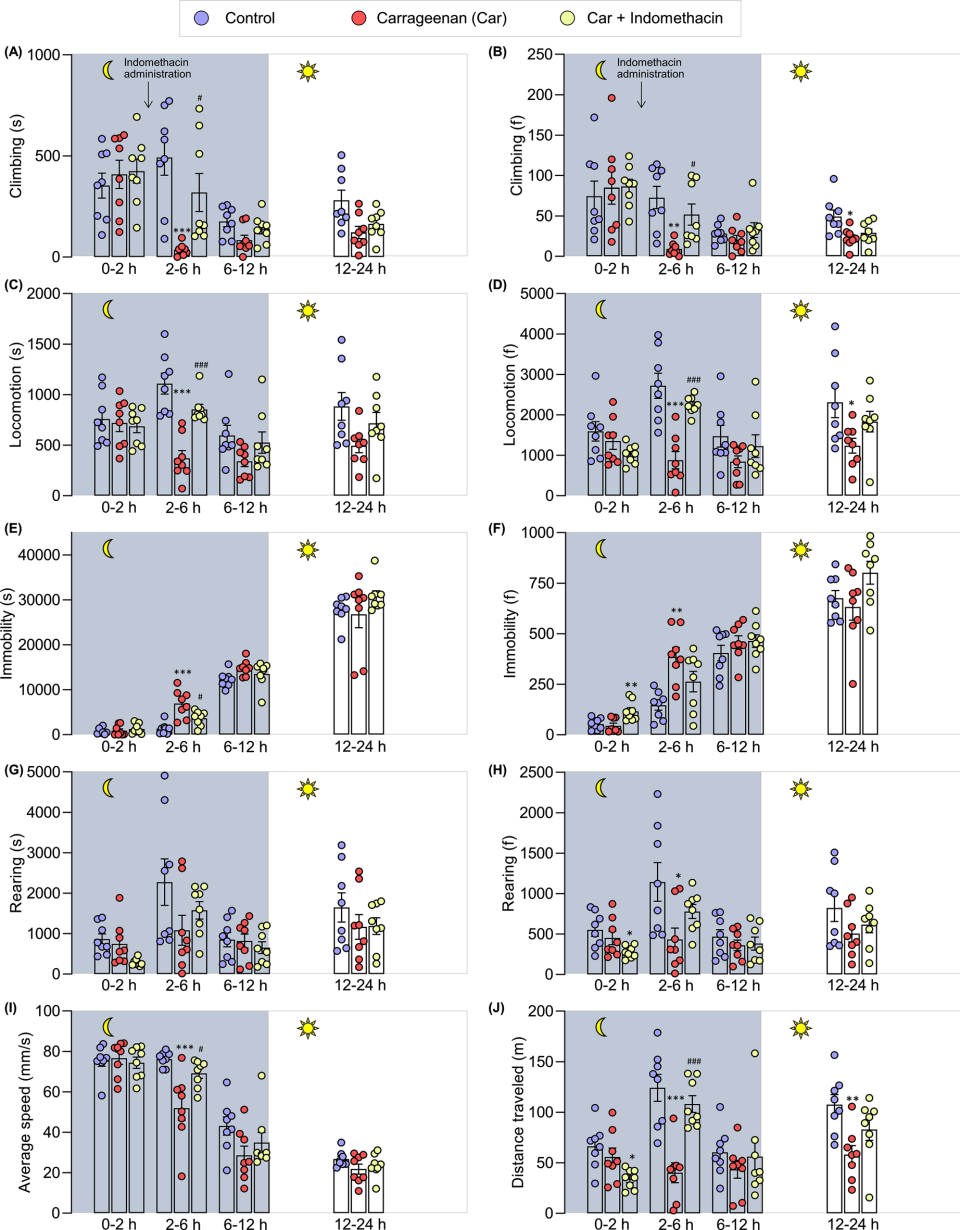
Long-term locomotor activity of control-, carrageenan-, carrageenan + indomethacin-treated mice in automated home-cage LABORAS. Long-term locomotor activity is demonstrated in four sections: 0–2 h (exploratory behaviors), 2–6 h (therapeutically effective duration of indomethacin at nighttime), 6–12 h, and 12–24 h (therapeutically ineffective duration of indomethacin at nighttime). The symbol of the moon indicates the nighttime and the sun for the daytime. Data are expressed as means ± SEM from 8 mice in each group. *, **, *** denote significant difference compared to the control group at p < 0.05, p < 0.01, p < 0.001, respectively. ^#^ and ^###^ denote significant difference compared to indomethacin-treated group at p < 0.05, p < 0.001, respectively.

The behavioral data were divided into four sections to define the effect of carrageenan on the locomotive behavior of the mice: 0–2 h (dark), 2–6 h (dark), 6–12 h (dark), and 12–24 h (light) post-carrageenan administration. The time points of 0–2, 2–6, 6–12, and 12–24 h represented exploratory behaviors (2 h), duration when indomethacin was therapeutically effective at night (4 h), duration when indomethacin was therapeutically ineffective at night (6 h), and post-drug administration behaviors during daytime (12 h) (Figs. 1D, 8). These time points covered daytime and nighttime conditions in a 12 h–12 h light–dark cycle. At 0–2 h post-carrageenan administration, the mobile behaviors were not substantially differed among the three animal groups: control, carrageenan, and carrageenan + indomethacin. However, the frequency of immobility, rearing, and distance traveled between the control and carrageenan + indomethacin groups significantly differed. At this time point, the mice in the carrageenan + indomethacin group were not administered with indomethacin. Hence, the behavioral data differed from the control group possibly because of the effects of carrageenan. From 2 to 6 h duration, total locomotion was determined and used to evaluate the efficacy of indomethacin at nighttime when indomethacin was therapeutically effective. The results showed that the carrageenan-treated group spent less time on mobile behaviors and more time on immobility compared with that of the control group. The carrageenan-treated group also revealed the following traits compared with those of the control group: less frequent mobile behaviors and more frequent immobility, less average speed, and distance traveled. Moreover, indomethacin treatment improved all the behaviors impaired by carrageenan as indicated by the improved frequency and duration of behaviors. The duration and frequency of climbing, locomotion, immobility, average speed, and distance traveled of the treated mice significantly differed from those of the carrageenan-induced mice. From 6 to 12 h, when indomethacin was therapeutically ineffective, the carrageenan-treated mice spent lesser time on mobile behaviors and longer time on immobility than the control- and indomethacin-treated mice. The carrageenan-treated mice also demonstrated less frequent mobile behaviors and higher immobility compared with the two groups. However, only slight but statistically insignificant differences were observed between the groups during 6–12 h measurements.

The analysis of the locomotor activity during daytime for a total of 12 h showed no substantial difference between the control and carrageenan groups except locomotion frequency, rearing frequency, and distance traveled, which indicated that the measurement of long-term locomotive behaviors was not suitable during daytime. Therefore, this study revealed that the impairment of long-term locomotor activity of the carrageenan-treated group observed at nighttime significantly differed from that of the control group and this impairment could be improved by indomethacin (Fig. 8).

## Discussion

The failure of analgesic drug development is attributed to the complex and diverse pathological conditions of pain, lack of sensitivity of clinical trials, and insufficient preclinical pain models. Specifically, the preclinical evaluation of pain, is limited by the pathological conditions of rodents that do not fully mimic the pain condition in humans, insufficient measures of pain assessment, reproducibility, variable molecular pathologies between rodent models and human, and lack of well-validated molecular targets ^4,42^. A decreased locomotor activity is currently recognized as a measure of non-reflexive pain in numerous animal models to overcome the limitations associated with the poor preclinical assessment of pain^16–22^. In this study, a behavioral system was defined to evaluate short- (exploratory behaviors) and long-term locomotor activity of carrageenan-induced inflammatory mice in the automated home-cage environment. von-Frey and plantar tests revealed that carrageenan significantly decreased the reflexive pain behaviors and impaired short- and long-term locomotor activity. Interestingly, non-reflexive pain behaviors in the automated home-cage LABORAS were strongly correlated with reflexive pain behaviors. Carrageenan did not affect the motor coordination of mice, confirming that carrageenan-induced locomotor deficits were pain-related, not motor-related. Furthermore, the effect of a standard analgesic drug, e.g., indomethacin, on these particular manifestations showed promising results. In particular, indomethacin improved mobile behaviors and decreased immobility. Therefore, our findings demonstrated the potential use of non-reflexive pain behaviors in pharmacological screening.

An inflammatory response is widely implicated as the primary mechanism of inflammatory pain. Carrageenan-induced rodents are commonly used to model inflammatory pain and considered clinically relevant. In the current study, carrageenan was used to induce pain-like behaviors presented as the decreased sensitivity to mechanical and thermal stimuli and impaired exploratory and locomotive behaviors. The carrageenan model was chosen because of its ability to activate the immune response in peripheral and central nervous systems^43,44^. At the mechanistic levels, an excessive amount of inflammatory mediators, including cytokines, chemokines, and PGE2, was found in the paw tissues and spinal cord of mice^46–46^. In the modulation of synaptic transmission, carrageenan induces inflammatory pain by modulating changes in spinal nociception^47^. In the spinal cord, carrageenan modulates the glutamatergic system via metabotropic glutamate receptors (mGluRs), AMPA, and kainate GluR5 receptors^48,49^. Despite the glutamatergic system, the spinal GABAergic system is modulated after carrageenan is peripherally injected, thereby affecting pain transmission^50^. The involvement of nociceptors, including vanilloid receptor 1 and transient receptor potential A 1 (TRPA1) channels, is reported in carrageenan-induced hyperalgesia^53–53^. Previous studies on the activity of carrageenan on pain behaviors showed its ability to induce thermal and mechanical hyperalgesia^43,44^. In line with previous studies, our research found that carrageenan-induced mechanical and thermal hypersensitivities initially occurred at 2 h, peaked at 6 h, and remained for 24 h. The expression of proinflammatory mediators (TNF-α and IL-6) in paw and spinal cord tissues increased. These results indicated the potential involvement of carrageenan challenge on the peripheral and central mechanisms of inflammatory pain (Supplementary Fig. S2). Moreover, changes in locomotor activity after carrageenan induction are also reported. The locomotor activity was indicated by distance traveled, and speed decreased after the intraplantar (i.pl.) administration of carrageenan^21,22^. Consistently, in the present study, carrageenan decreased the exploratory behaviors and long-term locomotor activity of mice. These findings indicated that inflammatory pain induced by carrageenan led to a substantial decrease in locomotor activity, manifested by a drastic decline in mobile behaviors and increased immobility. Moreover, our results showed that the short-and long-term locomotor activity in the LABORAS, significantly reduced, and the rotarod test revealed no effects on motor coordination. This finding suggested that the impaired locomotor activity of the carrageenan-induced mice in home-cage monitoring was mainly pain-related, not motor-related. However, a previous study on spared nerve-injured (SNI) mice found SNI-induced motor deficits, which might limit its use^54^. The pain-related impairment in locomotion obtained with this model would increase the potential use of this model as a validated preclinical model of pain. Interestingly, in the present study, evoked and non-evoked pain behaviors showed a strong correlation. Hence, the use of evoked and non-evoked pain behaviors for the preclinical evaluation of drug candidates could increase the translational value of preclinical data to the clinical presentation of pain.

Through automated home-cage monitoring locomotor activities (climbing, locomotion, rearing, and immobility), along with distance traveled and average speed, can be specifically assessed. This automated machine provides more sensitive measures and details while keeping rodents in a naturalistic environment^38^. Measuring exploratory behaviors by automated home-cage monitoring has distinct advantages over classical locomotor activity test, the open-field test regarding data acquisition, environmental stress factors and sensitivity. LABORAS allows the acquisition of diverse behavioral data, including the duration and frequency compared to the open-field test. Moreover, in LABORAS, mice are kept in a home-like environment, which reduces the environmental stress compared to the open-field. Further, the higher sensitivity of LABORAS to detect vertical activity (rearing behavior) over the open-field test (infrared actimeter) has been reported^28^. The findings of this study indicated that the duration of locomotion and distance traveled were higher in the open-field test than in the LABORAS. This difference could be due to the larger size of the open-field test arena, where the sizes of the LABORAS cage and open-field test were 22 × 16 × 14 cm and 50 × 50 × 50 cm, respectively. Moreover, the environmental conditions also influence the exploratory behaviors of mice. The LABORAS cage provided a more home-like environment to mice compared with that of the open-field arena. Consequently, the exploratory behaviors associated with the novel environment might be reduced. Carrageenan impaired locomotive behaviors in LABORAS measurements to a greater extent compared with that in the open-field measurements. However, the difference could be due to the presence of sawdust in the LABORAS cage. The sawdust in the cage might affect the locomotor activity of carrageenan-induced mice because it could generate a mechanical stimulus on the paws. However, further experiments are required to evaluate the effect of bedding materials on the locomotive behavior of carrageenan-induced mice in the LABORAS environment.

Previous studies demonstrated that the open-field test should be combined with the LABORAS automated home-cage to provide a more detailed and comprehensive behavioral analysis of locomotor activities^55,56^. The LABORAS automated home-cage for the locomotion test has been used to characterize locomotor activities in several diseases in rodents, such as Alzheimer’s disease^26^, anxiety^27^, CNS-associated disorders^29,30^, as well as to evaluate the side effects of drugs on the CNS^28^. Therefore, for the first time, this study reported the ability to assess locomotor impairment in carrageenan-induced inflammatory mice in a home-cage system. Here, a longer behavioral observation in the automated home-cage could provide a more appropriate model for the pharmacological evaluation of drugs. Thus, the locomotor activity of carrageenan-induced rodents could be precisely assessed and be further used to investigate novel analgesic drugs. The assessment of pain by exploratory behaviors and long-term locomotor activity in the automated home-cage system might have further advantages over conventional methods of pain assessment.

Mice are identified as nocturnal rodents, so their day and night cycle is opposite to that of humans. Mice are more active at nighttime and they tend to stay inactive/sleep during the daytime^57^. Therefore, in this study, carrageenan was administered at nighttime (18.00 h) to distinguish behavioral measures between treatment groups. As expected, all mobile behaviors, including climbing, locomotion, rearing, and distance traveled of the mice administered with carrageenan at nighttime were impaired compared with those during daytime. Moreover, the mobility of the control group was higher at nighttime than that during the daytime. This finding was consistent with previous results, which demonstrated the nocturnal behaviors of mice.

Several automated home-cage behaviors have been identified to evaluate the innate behaviors of rodents in a home-like environment. The technology of automated home-cage monitoring includes video analysis, radio frequency identification (RFID) technology, and vibration/force signal from the surface of the cage. Each system has its advantages and disadvantages^38,58^. For example, although LABORAS can be used to characterize behavioral changes in several disease models, this system can only track a single mouse per cage during an experiment. Isolation of mice may affect their general well-being, physical activity, and behaviors, such as body weight^59^, stress^60^, and cognitive performance^61^ because mice are social creatures. In addition, social isolation may contribute to the translation of behavioral pain because pain modulation in mice can be influenced by social factors^59,62^. Furthermore, some behaviors detected in the LABORAS remain undefined, thereby limiting the further interpretation of results. Another limitation of the present study is the simultaneous use of only two cages. However, one control unit of LABORAS supports the simultaneous acquisition of data in eight platforms. Hence, the maximum possible number of animals per time should be used in the analysis to limit the variability of results. Moreover, the age of mice used in this study (6–8 weeks) resembles the puberty stage in humans^63,64^, yet clinically pain is more common in elderly patients^65^

Indomethacin is a major analgesic drug commonly prescribed to treat inflammatory pain. Hence, indomethacin which is an efficacious cyclooxygenase-2 (COX-2) inhibitor clinically used in humans was selected to validate this model through pharmacological intervention^67^. At the mechanistic level, indomethacin inhibited proinflammatory mediators, including TNF-α and IL-6, in paw and spinal cord tissues (Supplementary Fig. S2). In addition, indomethacin was selected over other analgesic drugs because it did not affect locomotor activity and motor coordination^66^  as well as its prominent anti-inflammatory activity. As shown in this study, indomethacin did not affect the general behavior of mice in the automated home-cage LABORAS (Fig. 1F and Supplementary Fig. S3). Gabapentinoids and morphine, which are other commonly used analgesics decrease and increase the locomotive behaviors, respectively^68,69,70^. In one study, indomethacin increased exploratory behaviors in LPS-induced mice, as indicated by their increased rearing behaviors and distance traveled^71^. Consistent with the previous work, our study revealed that indomethacin improved the locomotor activity in carrageenan-induced mice in short- and long-term periods of monitoring. The results demonstrated that indomethacin increased mobile behaviors while decreasing immobility, indicating an improvement in locomotor activity. This study provides insights into the effects of indomethacin on the improvement of locomotor activity in carrageenan-induced mice. Altogether our study evidences the potential use of this model for the pharmacological screening of analgesic drugs.

## Conclusion

In conclusion, our results suggested that the locomotor activity of mice could be impaired by carrageenan treatment, but this impairment could be reversed by indomethacin administration. Automated behavioral analysis provided a comprehensive and detailed description of pain-related locomotive behaviors in a carrageenan-induced inflammatory mouse model at different time frames.

## Methods

### Animals

Male ICR mice weighing 18–25 g were purchased from Nomura Siam International Co., Ltd., Bangkok, Thailand, and acclimatized for ± 2 weeks before being used in the experiments. The mice were given free access to food and water and maintained under the following conditions: 12–12 h light–dark cycle, 50%–60% humidity, and ± 25 °C. They were housed in standard static filtered top cages, and five mice were enclosed in each cage. Mice (6–8 weeks) from different cages were randomly assigned to each test and treatment. All the protocols were approved by the Institutional Animal Care and Use Committee (IACUC) of the Faculty of Pharmaceutical Sciences, Chulalongkorn University (protocol No. 2033004) and performed in accordance with the recommendations of the IACUC. The weight and health of the mice were monitored daily. All the tests were in compliance with the ARRIVE guidelines (Animal Research: Reporting of In Vivo Experiments).

### Carrageenan induction and experimental timeline

The mice were injected with 50 µL of 1% carrageenan (w/v) in a saline solution (Sigma, St. Louis, MO, USA) into the ipsilateral paw (left hind paw) via a 30-gauge needle. Behavioral analysis on evoked and non-evoked pain was performed using von Frey and plantar tests and LABORAS automated home-cage monitoring, respectively. The mice were divided into three groups: naïve mice treated with a vehicle (0.5% carboxymethyl cellulose (CMC); vehicle-control), carrageenan-induced mice treated with the vehicle (disease control), and carrageenan-induced mice treated with indomethacin (positive control). Evoked pain behavior was measured 2, 4, 6, and 24 h post-carrageenan administration. Short- and long-term non-evoked pain measures were obtained.

### Pharmacological intervention

Indomethacin was used as pharmacological intervention to characterize the classically used carrageenan-induced inflammatory pain model. Indomethacin (Sigma-Aldrich, St Louis, MO, USA) was suspended in 0.5% (w/v) CMC in saline and administered orally at a constant volume of 10 ml/kg. Behavioral evaluation was split into two different cohorts. One cohort was examined via mechanical and thermal stimuli-evoked behavioral tests and short-term exploratory behavioral analysis. The mice were administered with indomethacin or vehicle (0.5% CMC) 2 h after they were induced with carrageenan. Short-term locomotor activity, von Frey test, and plantar tests were performed 1, 2, and 3 h after the treatment. The long-term locomotor activity of the second cohort was analyzed for 24 h. The mice were administered with carrageenan or vehicle (normal saline) and placed in LABORAS home cages. After 2 h, they were taken out of their cages and administered either with 0.5% CMC or 10 mg/kg indomethacin. Behavioral measures were obtained continually for 24 h.

### Evoked pain assessment through von Frey and plantar tests

von Frey and plantar tests were performed to assess mechanical and thermal sensitivities, respectively. The mice were acclimatized into a behavioral testing apparatus for approximately 1 h before behavioral assessments. In the von Frey test, a series of von Frey filaments (Serial No. 18011, Stoelting, Wood Dale, IL, USA) was applied five times to the ipsilateral paw of the mice in an ascending order of forces (1.65–5.07 g). Hind paw lifting, shaking, and licking were considered a positive response. The minimum force of the filament that induced three positive responses out of five consecutive applications (response frequency ≤ 60%) was considered the paw withdrawal threshold (cut off = 5.07 g). Then, the mice were transferred to a plantar test apparatus (Ugo-Basile, Varese, Italy). After 1 h, radiant heat (90 IR units) was applied to the ipsilateral paw of the mice. The latency from the onset of heat application to the lifting of the paw was set as the paw withdrawal latency. Measurement was assessed repeatedly at least three times in 5 min of intervals. The cut-off of latency was set at 18 s to avoid tissue damage.

### Locomotor activity assessment through automated-behavioral analysis

LABORAS automated behavioral technology (Metris, Hoofddorp, Netherlands) was used. Through this technology, behavior was defined on the basis of vibration and force signal detection generated by an individual mouse in a cage and processed via the LABORAS program. The locomotor activity of the mice was characterized and distinguished into several locomotive behaviors, such as rearing, climbing, walking, running, immobility, average speed, and distance traveled. Locomotor activities driven by exploratory behaviors and long-term locomotor activities were evaluated as a reading of behavioral pain. Each LABORAS cage was provided with sawdust and corn-cob bedding (B&C Pulaski Corp. ltd, Thailand), had a granular shaped, and had a size of approximately 1/8 inch to fulfill environmental enrichment. The test was performed for 30 min and 24 h to examine exploratory behaviors and long-term locomotor activity, respectively. Short-term locomotor activities were evaluated in the daytime, whereas long-term locomotor activities were assessed during night and day times. The mice from each cage were randomly assigned to control, carrageenan, or carrageenan + indomethacin groups. Two cages of LABORAS were used to analyze two different parallel treatments. For the experiment, two mice from different treatments, i.e., control, carrageenan, or indomethacin groups, were measured simultaneously for each day. Before the actual test, the LABORAS automated behavioral system was calibrated with the reference weight. In the test session, the mice were individually placed in a type II cage with a size of 22 cm × 16 cm × 14 cm. Then, short- and long-term behavioral measures were automatically quantified in behavioral duration (s) and behavioral frequency (counts). The locomotor activity was translated into mobile behaviors (climbing, rearing, and locomotion, including walking and running), immobility, unidentified behaviors (behaviors except those behaviors mentioned previously), distance traveled, and average speed. The undefined behaviors were excluded from the study.

### Open-field test

An open-field test, which is a classical locomotor activity test, was performed to determine locomotor activities driven by the exploratory behaviors of the mice in a novel environment. The mice from different cages were randomly assigned to each treatment. They were orally administered with either vehicle (0.5% CMC) or 10 mg/kg indomethacin after 2 h of carrageenan injection. After 1 h, the responses of the mice from each group to open-field exposure were assessed for 30 min. The mice were individually placed in an open-field arena (50 cm × 50 cm × 50 cm), and their movements were quantified and tracked using a VideoMOT2 (TSE systems, Bad Homburg, Germany).

### Rotarod test

The effect of the peripheral injection of carrageenan on motor coordination was evaluated with a rotarod test. A constant rotation speed of 17 rpm was set, and the test was performed for 3 min^72^. The time that the mice could remain on the rotating rod was recorded and considered the rotarod latency.

### Statistical analysis

Data were expressed as mean ± standard error of mean (S.E.M.). They data were analyzed and visualized by GraphPad Prism 9 (GraphPad Software Inc., La Jolla, CA, USA). For comparison between groups, the differences between groups were explored through ANOVA followed by the Bonferroni post hoc test. Data were considered statistically significant when the probability value (p) was less than 0.05.

## Supplementary Information

Supplementary Information.

## Data Availability

Data will be made available upon request. Contact pasarapa.c@chula.ac.th.
