# Supplementary material for: Automated home-cage for the evaluation of innate non-reflexive pain behaviors in a mouse model of inflammatory pain
Source: Sci Rep. 2021 Jun 10;11:12240. doi: 10.1038/s41598-021-91444-4 (PMC8192791; doi:10.1038/s41598-021-91444-4)
Supplement: Supplementary file 1 — Supplementary Information. [file 41598_2021_91444_MOESM1_ESM.docx]

**Automated home cage for the evaluation of innate non-reflexive pain behaviors in a mouse model of inflammatory pain**

Hasriadi^1^, Peththa Wadu Dasuni Wasana^1^, Opa Vajragupta^2^, Pornchai Rojsitthisak^3,4^ & Pasarapa Towiwat^4,5*^

1*Pharmaceutical Sciences and Technology Program, Faculty of Pharmaceutical Sciences, Chulalongkorn University, 10330, Bangkok, Thailand*

2*Research affairs, Faculty of Pharmaceutical Sciences, Chulalongkorn University, 10330, Bangkok, Thailand*

3*Department of Food and Pharmaceutical Chemistry, Faculty of Pharmaceutical Sciences, Chulalongkorn University, 10330, Bangkok, Thailand*

4*Natural Products for Ageing and Chronic Diseases Research Unit, Chulalongkorn University, Bangkok 10330, Thailand*

5*Department of Pharmacology and Physiology, Faculty of Pharmaceutical Sciences, Chulalongkorn University, 10330, Bangkok, Thailand*

**The effect of carrageenan on exploratory behaviors in the LABORAS system**

To provide better comparisons among three groups (control, carrageenan, carrageenan + indomethacin groups) in exploratory behaviors, the cumulated locomotor activity at each time point is expressed as the average of five-minute intervals for 30 minutes, with sections of 0-5 min, 5-10 min, 10-15 min, 15-20 min, 20-25 min, and 25-30 min. The results indicated that the effects of carrageenan differed slightly from those of control and indomethacin groups during the first 0-5 min as indicated by a significant difference in only distance traveled. Furthermore, a considerable difference between the carrageenan and other groups was first observed at 5-10 min, with a significant difference in duration and frequency of locomotion and distance traveled **(Supplementary Figure S1)**.


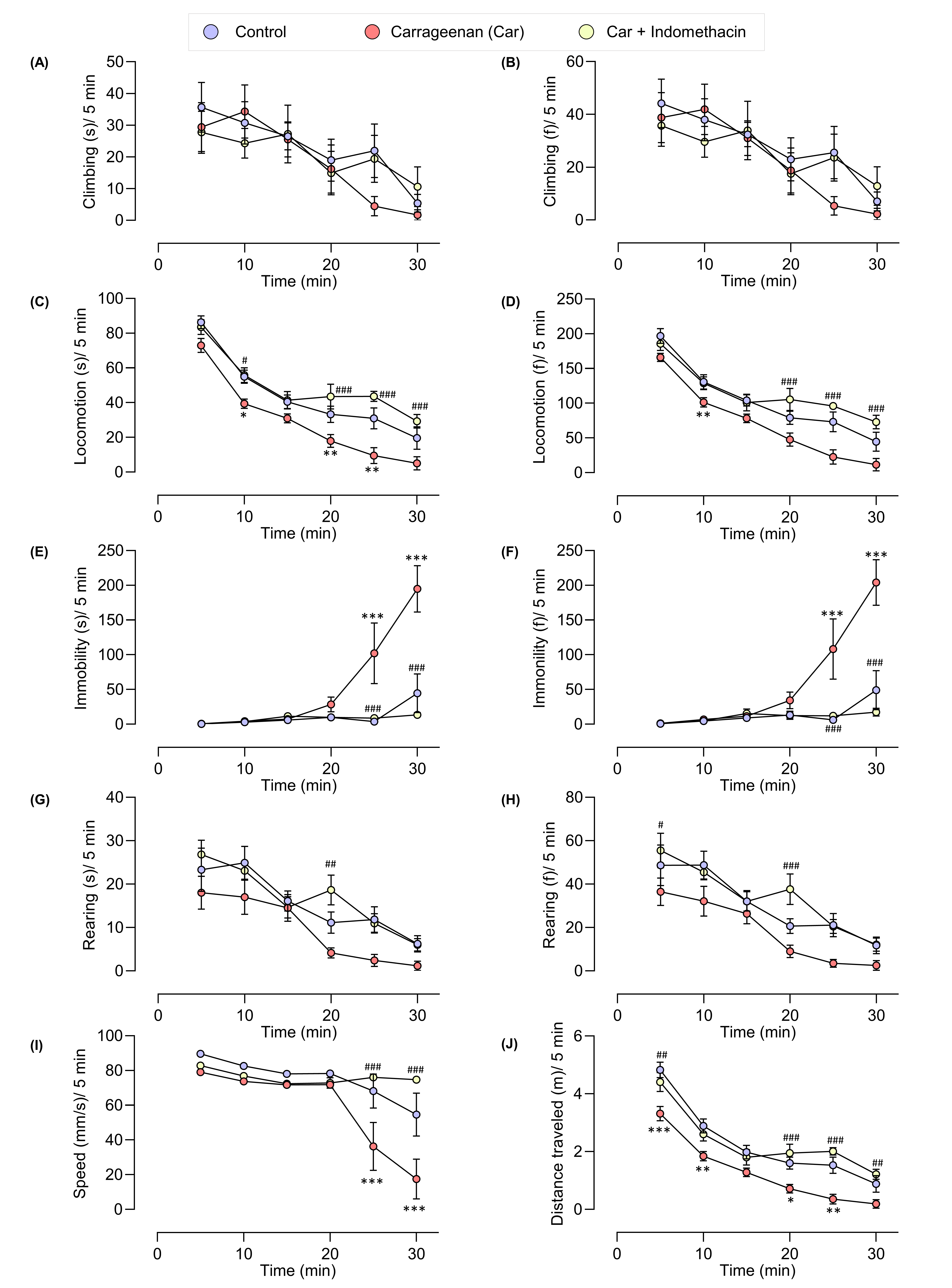


**Supplementary Figure S1.** **Exploratory behaviors of control mice, carrageenan mice, carrageenan + indomethacin mice in automated- cage LABORAS per each five minutes of post-carrageenan administration.** Data are expressed as means ± SEM from 8 mice in each group. *, **, *** denote significant difference compared to the control group at p<0.05, p<0.01, and p<0.001, respectively. ^##^ and ^###^ denote significant difference compared to indomethacin-treated group at p< 0.01, p< 0.001, respectively.

**Potential effect of carrageenan on the peripheral and central mechanisms of inflammatory pain**

Proinflammatory mediators, including TNF-α and IL-6, mediate peripheral and central sensitization in pain transmission. The expression levels of TNF-α and IL-6 in the paw tissues and spinal cords of mice were analyzed to investigate whether the intraplantar injection of carrageenan induces pain through the peripheral and central mechanisms of inflammatory pain. After behavioral analysis, the mice were euthanized via CO_2_ asphyxiation followed by cervical dislocation. Then, their paw tissues and spinal cords were isolated, extracted in cold PBS, sonicated, and centrifuged at 10,000 rpm. After centrifugation, the supernatant was collected, and two proinflammatory mediators, TNF-α and IL-6, were analyzed with ELISA in accordance with the manufacturer’s instructions (BioLegend, San Diego, CA, USA).

Carrageenan significantly increased the expression levels of TNF-α and IL-6 in the paw tissues and the spinal cord compared with those of the control group. However, indomethacin treatment significantly reduced the carrageenan-induced expression of TNF-α and IL-6 in both tissues **(Supplementary figure S2)**. These data demonstrated that carrageenan administration induced pain-like behaviors through the peripheral and central mechanisms of inflammatory pain, and the expression of TNF-α and IL-6 could be inhibited by indomethacin administration.


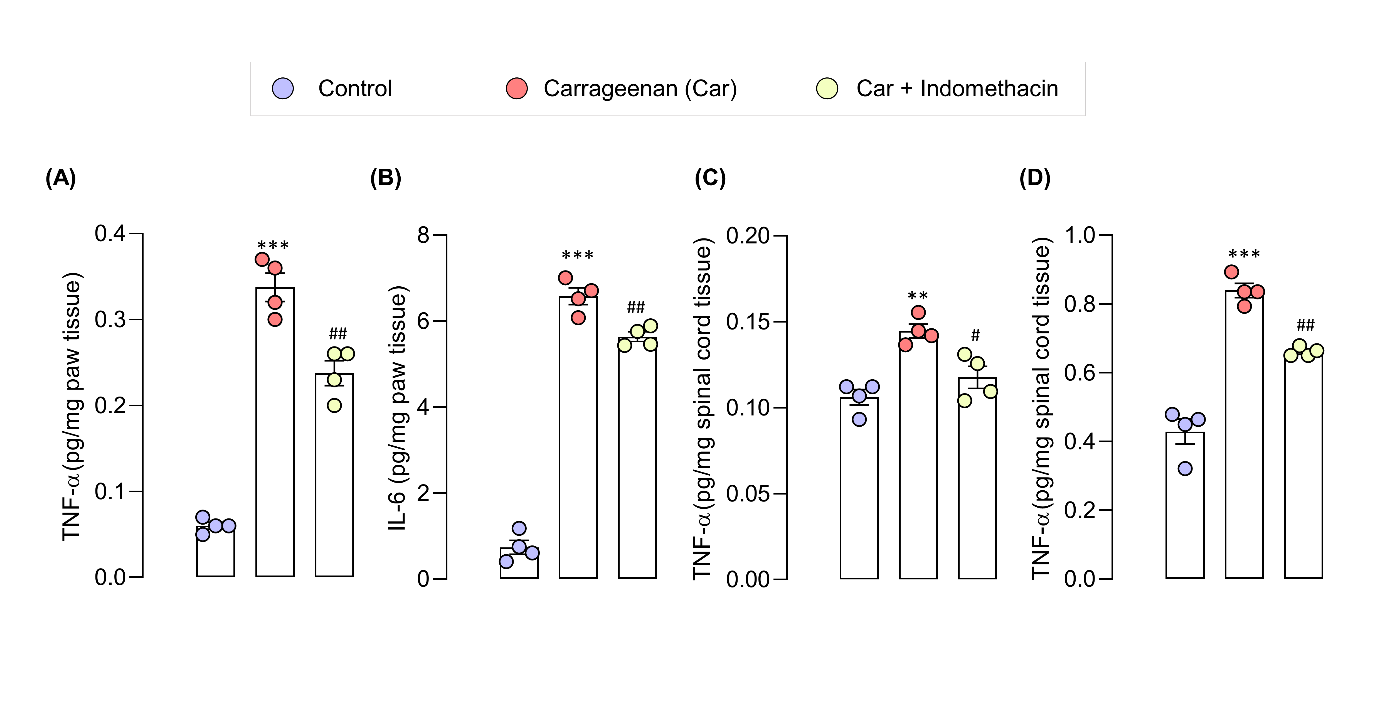
**Supplementary Figure S2. The effect of carrageenan on cytokines expression in the paw tissue and spinal cord of mice.** Data are expressed as means ± SEM (n = 4). ** and *** denote significant difference compared to the control group at p<0.01, and p<0.001, respectively. # and ## denote significant difference compared to indomethacin-treated group at p< 0.05, p< 0.01, respectively.

**The effect of indomethacin on general behaviors**

The general behaviors of mice after indomethacin administration were measured using the LABORAS system. Mice were treated with indomethacin, and the vehicle (0.5% CMC), randomly selected from different cages, were individually placed in the LABORAS cage, and the behaviors were recorded for 24 h. Our result indicated no difference in all behaviors evaluated between indomethacin-treated mice and control mice **(Supplementary Figure S3)**. This result demonstrated that no substantial effects of indomethacin on general behaviors.


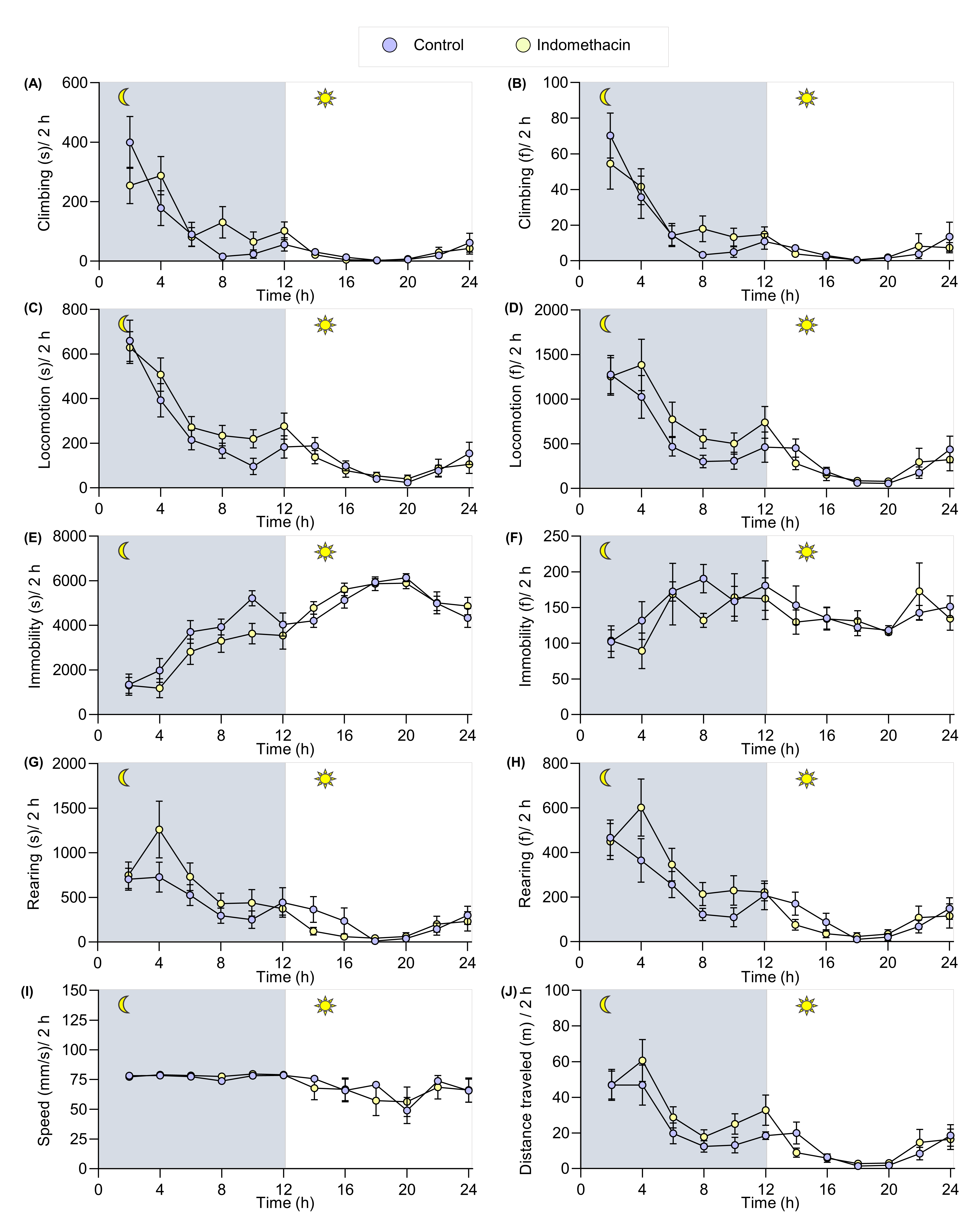


**Supplementary Figure S3.** **The effect of indomethacin on general behaviors in automated- cage LABORAS.** Data are expressed as means ± SEM from 8 mice in each group.
